# Induction of the type I interferon response in neurological forms of Gaucher disease

**DOI:** 10.1186/s12974-016-0570-2

**Published:** 2016-05-12

**Authors:** Einat B. Vitner, Tamar Farfel-Becker, Natalia Santos Ferreira, Dena Leshkowitz, Piyush Sharma, Karl S. Lang, Anthony H. Futerman

**Affiliations:** Department of Biomolecular Sciences, Weizmann Institute of Science, Rehovot, 76100 Israel; Bioinformatics Unit of The Biological Services Department, Weizmann Institute of Science, Rehovot, 76100 Israel; Institute of Immunology, Medical Faculty, University Duisburg-Essen, Essen, Germany; Department of Gastroenterology, Hepatology and Infectious Diseases, Heinrich-Heine-University Düsseldorf, Düsseldorf, Germany; Present address: Department of Infectious Diseases, Israel Institute for Biological Research, Ness-Ziona, 74100 Israel; Present address: Synaptic Function Section, The Porter Neuroscience Research Center, National Institute of Neurological Disorders and Stroke, National Institutes of Health, Bethesda, MD 20892 USA

## Abstract

**Background:**

Neuroinflammation is a key phenomenon in the pathogenesis of many neurodegenerative diseases. Understanding the mechanisms by which brain inflammation is engaged and delineating the key players in the immune response and their contribution to brain pathology is of great importance for the identification of novel therapeutic targets for these devastating diseases. Gaucher disease, the most common lysosomal storage disease, is caused by mutations in the *GBA1* gene and is a significant risk factor for Parkinson’s disease; in some forms of Gaucher disease, neuroinflammation is observed.

**Methods:**

An unbiased gene profile analysis was performed on a severely affected brain area of a neurological form of a Gaucher disease mouse at a pre-symptomatic stage; the mouse used for this study, the *Gba*^flox/flox^; nestin-Cre mouse, was engineered such that *GBA1* deficiency is restricted to cells of neuronal lineage, i.e., neurons and macroglia.

**Results:**

The 10 most up-regulated genes in the ventral posteromedial/posterolateral region of the thalamus were inflammatory genes, with the gene expression signature significantly enriched in interferon signaling genes. Interferon β levels were elevated in neurons, and interferon-stimulated genes were elevated mainly in microglia. Interferon signaling pathways were elevated to a small extent in the brain of another lysosomal storage disease mouse model, Krabbe disease, but not in Niemann-Pick C or Sandhoff mouse brain. Ablation of the type I interferon receptor attenuated neuroinflammation but had no effect on GD mouse viability.

**Conclusions:**

Our results imply that the type I interferon response is involved in the development of nGD pathology, and possibly in other lysosomal storage diseases in which simple glycosphingolipids accumulate, and support the notion that interferon signaling pathways play a vital role in the sterile inflammation that often occurs during chronic neurodegenerative diseases in which neuroinflammation is present.

**Electronic supplementary material:**

The online version of this article (doi:10.1186/s12974-016-0570-2) contains supplementary material, which is available to authorized users.

## Background

Type I interferons (IFNs; Ifnα1-13 and Ifnβ), a large family of structurally related cytokines, are key components of the innate immune response and are the fundamental cellular defense mechanism against viral infection [1]. IFNs are currently used therapeutically for a number of viral diseases, for numerous malignancies, and for a number of chronic inflammatory disorders such as the demyelinating disease and multiple sclerosis [2]. However, even though activation of the type 1 IFN response has been intensely studied as part of the host response to viral and bacterial infection, induction of this response has also been observed in the absence of infection [3]. In the central nervous system (CNS), IFN activation occurs in amyotrophic lateral sclerosis (ALS) [4], in Alzheimer’s disease (AD) [5], during aging [6], in Aicardi-Goutières syndrome (AGS) [7, 8], and upon axonal transection [9]. Activation of the IFN response under sterile conditions has led to a paradigm shift in our understanding of the role of these cytokines and their role in inflammation inasmuch as the type I interferonopathies are now believed to comprise a heterogeneous group of genetically determined diseases characterized by inappropriate activation of the type I IFN response [10].

A wide range of neurodegenerative conditions are characterized by brain inflammation, including Alzheimer’s and Parkinson’s diseases (PD) [11], and the less common lysosomal storage diseases (LSDs) [12–14], which are normally caused by the defective activity of a lysosomal hydrolase. One such LSD is Gaucher disease (GD), caused by mutations in the *GBA1* gene, which encodes the lysosomal enzyme, acid-β-glucosidase (glucocerebrosidase, GCase) [15], resulting in the accumulation of the sphingolipid glucosylceramide (GlcCer) and its deacylated form, glucosylsphingosine (GlcSph) [16, 17]. GD is the most common LSD and ~10 % of GD patients worldwide present with neurological symptoms. However, the distinction between neurological and non-neurological forms of the disease has become blurred over the recent years based on the finding that heterozygous mutations in *GBA1* are a major risk factor for PD [18]. Thus, understanding the pathological pathways activated upon alteration of GCase activity in the brain might have significant ramifications for understanding more common neurodegenerative diseases such as PD.

The neuronopathic forms of GD, types 2 and 3, are characterized by astrogliosis, neuronophagia (i.e., brain inflammation), and neuronal loss [19, 20]. We now demonstrate elevation of IFNβ levels in neurons and activation of the type 1 IFN response in mouse models of neuronopathic Gaucher disease (nGD), but to a much lower extent, or absent completely, in other LSD mouse models. This discovery was made during an unbiased gene profile analysis of a severely affected brain area of an nGD mouse at a pre-symptomatic stage. The mouse used for this study, the *Gba*^flox/flox^; nestin-Cre mouse [21], was engineered such that *GBA1* deficiency is restricted to cells of neuronal lineage, i.e., neurons and macroglia. Robust induction of type I IFN-stimulated genes (ISGs), including pathogen recognition receptors (PRRs) and antiviral genes was observed; the lack of activation of this pathway in other LSDs suggests that GlcCer and/or GlcSph specifically activate the antiviral response. Moreover, our data suggest that a key player in the pathway of programmed necrosis, the protein mixed lineage kinase domain-like (MLKL), is a novel ISG which is induced down-stream to the IFN receptor in cells of myeloid lineage. Together, our data demonstrate that the IFN response can be activated in neuroinflammation under sterile conditions and that this pathway might be involved in nGD pathogenesis, although whether this pathways is detrimental or beneficial remains to be established.

## Results

### Induction of the type I interferon response

**Fig. 1:**
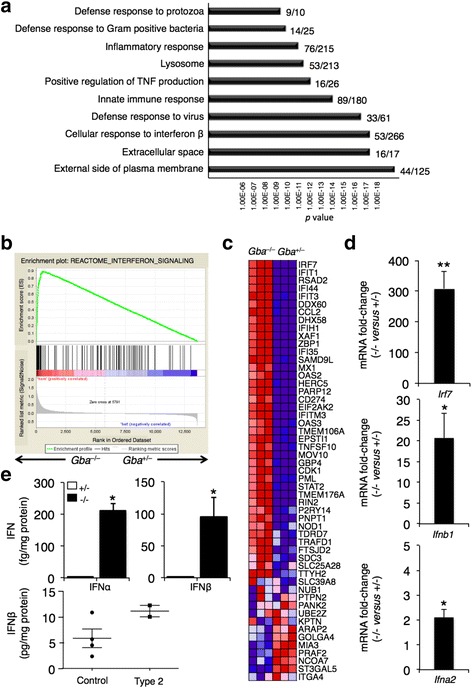
Activation of the type I IFN response in nGD. **a** The 10 most significantly enriched GO terms for up-regulated genes illustrating enrichment of type I IFN-related pathways. The plot shows the enrichment *p* values. The number of differentially expressed genes out of the total number of genes included in the term detected by the array are indicated. *n* = 3, 14-day-old mice. **b**, **c** Gene set enrichment analysis (GSEA) of gene expression in *Gba* ^−/−^compared to *Gba* ^+/–^ mice. **b** GSEA enrichment plot. False discovery rate (FDR) *q* value; normalized enrichment scores (NES). **c** Heat map displaying the relative expression level of proteins in the interferon signaling set, sorted by their fold change. Depicted are the top 51 genes. **d** qPCR analysis of *Irf7* mRNA in the VPM/VPL obtained from 16-day-old mice and of *Ifnb1* and *Ifna2* mRNA in cortical tissues obtained from 21-day-old mice. **p < 0.001, *p < 0.005. Values are shown as fold change in mRNA levels (*Gba* ^−/−^ versus control) and are means ± s.e.m, *n* = 3–4. **e** ELISA analysis of IFNα and IFNβ protein levels in cerebral brain tissue obtained from 21-day-old control and *Gba* ^−/−^ mice. IFNα and IFNβ were not detected in control samples. *p < 0.005. Values are means ± s.e.m, *n* = 3 (upper panel). IFNβ protein levels in cerebellum of two human type 2 GD patients (*n* = 2) compared to age-matched control brains (*n* = 4). *p* value = 0.06 (*lower panel*)

To determine changes in gene expression in the brain of the *Gba*^flox/flox^; nestin-Cre mouse, herein referred to as the *Gba*^−/−^ mouse, we performed a microarray analysis on the ventral posteromedial/posterolateral (VPM/VPL) region of the thalamus, the area which is most affected in the *Gba*^−/−^ mouse, at 14 days of age, an age at which the first signs of neuroinflammation and neuronal loss are observed but prior to overt signs of disease [22]. Approximately 10 % of the genes detected by the array were differentially expressed (absolute fold-change ≥1.5, *p* value ≤0.05), with 907 genes up-regulated in *Gba*^−/−^ versus *Gba*^+/−^ mice and 481 genes down-regulated (for a complete list of the differentially-expressed transcripts, see Additional file 1: Table S1). Changes in expression of *Gfap*, *Lgals3*, *Kcnk4*, *Sptssb*, and *Ryr3* were confirmed by quantitative polymerase chain reaction (qPCR) using the same RNA samples used for the microarray analysis, and their fold-changes were ~100, ~230, ~0.35, ~0.35, and ~0.40, respectively. Up-regulated genes were next subjected to gene ontology (GO) analysis [23] (Fig. 1a). Among the up-regulated genes, innate immune response pathways, and surprisingly, type I IFN-related pathways, were highly enriched (Fig. 1a). The robust induction of the immune response, and in particular of the type I IFN response, was confirmed by the fact that the 10 most up-regulated genes were all genes that are induced during neuroinflammation, with most induced by type I IFN (Table 1).

**Table 1:** Top ten up-regulated genes

Gene symbol	Gene name	Fold change (−/− vs. +/−)	*p* value	Reference^a^
Cxcl10^*^ (IP-10)	Chemokine (C-X-C motif) ligand 10	86.3	<0.001	[68]
Usp18^*^	Ubiquitin specific peptidase 18	79.2	<0.005	[30]
Ifit1^*^	Interferon-induced protein with tetratricopeptide repeats 1	75.2	<0.001	[69]
Irf7^*^	Interferon regulatory factor 7	71.7	<0.005	[30]
Cxcl13^*^	Chemokine (C-X-C motif) ligand 13	67.2	<0.05	[70]
Gpnmb	Glycoprotein (Transmembrane) Nmb	63.0	<0.005	
Lcn2	Lipcalin 2	60.1	<0.005	
Oasl2^*^	2′-5′ oligoadenylate synthetase-like 2	56.0	<0.005	[30]
Tgm1	Transglutaminase 1, K polypeptide	46.5	<0.005	
Ifit3^*^	Interferon-induced protein with tetratricopeptide repeats 3	42.1	<0.001	[69]

Type I IFN-induced genes are indicated by asterisks

^a^References refer to studies demonstrating that these genes are induced by type I IFN

Gene set enrichment analysis (GSEA) confirmed that the gene expression signature of the *Gba*^−/−^ mouse was significantly enriched in IFN signaling genes, with an approximate false discovery rate of zero and a normalized enrichment score of 2.27 (Fig. 1b, c). Innate immunity is the first line of defense against infection and cell damage and is triggered by the recognition of pathogen/danger-associated molecular patterns (PAMPs or DAMPs) by PRRs. Genes encoding various PRRs were elevated in the *Gba*^−/−^ brain, including Toll-like receptors (*Tlr*), which encode transmembrane PRRs, other membrane-bound PRRs such as the C-type lectin receptors, *Clec7a* (Dectin-1) and *Clec5a* (MDL-1) [24], and the scavenger receptors *Cd36* and *Msr1* (SR-A1) [25] (Additional file 1: Table S1). Genes encoding cytosolic nucleic-acid sensing PRRs [26] were also up-regulated (Additional file 1: Table S1). PRR stimulation leads to IFN transcription and secretion [27] by interferon regulatory factor (IRF) transcription factors [28], of which *Irf7, Irf8, Irf9, Irf1*, and *Irf5* were up-regulated. *Irf7*, which is among the most highly-elevated genes (Table 1), is the master regulator of type I IFN signaling, and its expression is further up-regulated by type I IFN [29]. qPCR analysis confirmed that *Irf7* was highly induced in the VPM/VPL of *Gba*^−/−^ mice (Fig. 1d). Both *Ifnb1* and *Ifna2* mRNA levels were also elevated (~20 and ~2-fold, respectively) in the cerebral cortex of *Gba*^−/−^ mice (Fig. 1d), another highly affected brain area in nGD mice [22]. Moreover, IFNα and IFNβ were detected by enzyme-linked immunosorbent assay (ELISA) in cerebral tissue from *Gba*^−/−^ mice, but not from control mice (Fig. 1e). Crucially, levels of IFNβ were also elevated in the two available cerebella of human patients who succumbed to type 2 GD (Fig. 1e).

**Fig. 2:**
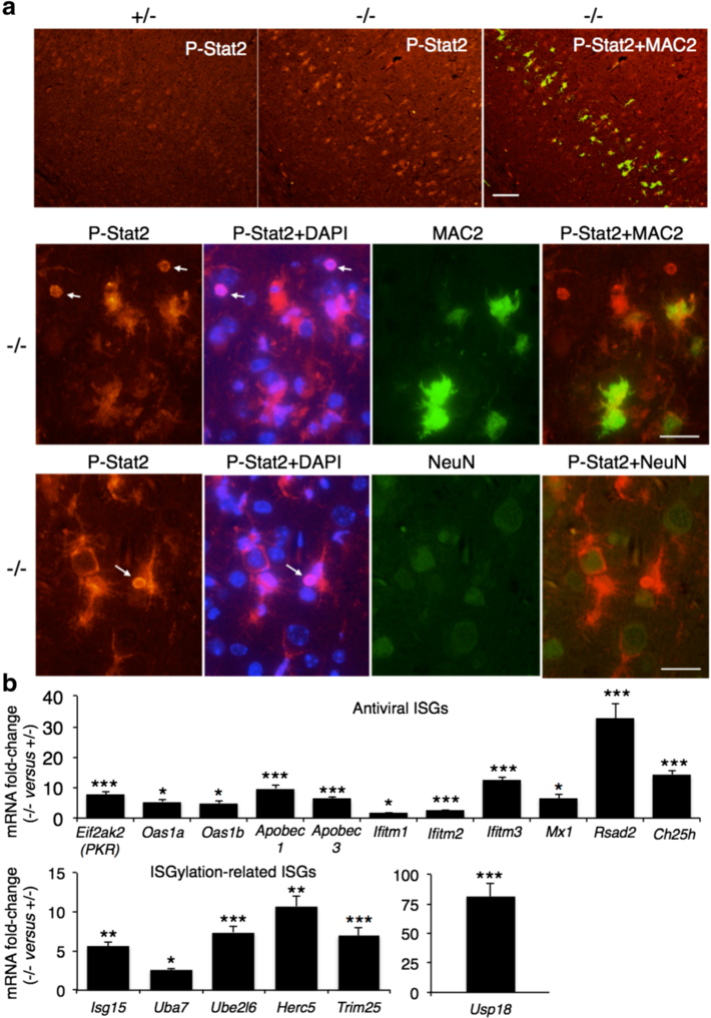
Induction of the JAK/STAT pathway in nGD. **a** P-Stat2 is increased in the nGD brain. Double immunofluorescence of cortical layer V in 16-day-old mice using anti-P-Stat2 (*red*) and anti-MAC2 (*green*, *upper* and *middle panels*) or anti-NeuN (*green*, *lower panel*) antibodies and the nuclear marker, DAPI (*blue*). *Arrows* indicate nuclear staining of P-Stat2. Scale bar, 25 μm. Results are representative of three experiments. **b** Microarray analysis of ISG expression in the nGD brain. Data is presented as fold change of mRNA levels (*Gba* ^−/−^ versus *Gba* ^+/–^). Values are means ± s.e.m, *n* = 3. *p < 0.05, **p < 0.05, ***p < 0.005. Janus kinase (JAK); signal transducer and activator of transcription (STAT)

IFNα and IFNβ bind the Ifnα/β receptor (IFNAR) to induces gene expression via the Janus kinase (JAK)-signal transducer and activator of transcription (STAT) pathway, resulting in expression of a large spectrum of ISGs [27]. Phosphorylated Stat2 was up-regulated in an affected *Gba*^−/−^ brain area (Fig. 2a), and its levels were increased in both MAC2-positive (i.e. activated microglia/macrophages) and MAC2-negative cells (Fig. 2a). P-Stat2 rarely co-localized with the neuronal nuclear marker NeuN, but neuronal cell bodies were frequently encircled by P-Stat2 staining, likely indicating P-Stat2 elevation in phagocytic cells (Fig. 2a). Finally, P-Stat2 staining was also detected in the nuclei of cells that were not labeled with the neuronal marker, NeuN (Fig. 2a).

A number of ISGs down-stream to JAK-STAT [30] were up-regulated, indicating activation of IFNAR and the antiviral response (Fig. 2b). Isg15*,* which mediates IFN-regulated ubiquitination of cellular and viral targets termed ISGylation [1], was up-regulated (Fig. 2b), as were genes encoding proteins which sequentially catalyze the conjugation of Isg15 to proteins [1] (Fig. 2b). ISGylation is reversible, and *Usp18*, a negative regulator of type I IFN signaling which catalyzes the hydrolysis of Isg15 [30], was one of the most highly up-regulated genes (Table 1 and Fig. 2b), consistent with a robust activation of the type I IFN response.

**Fig. 3:**
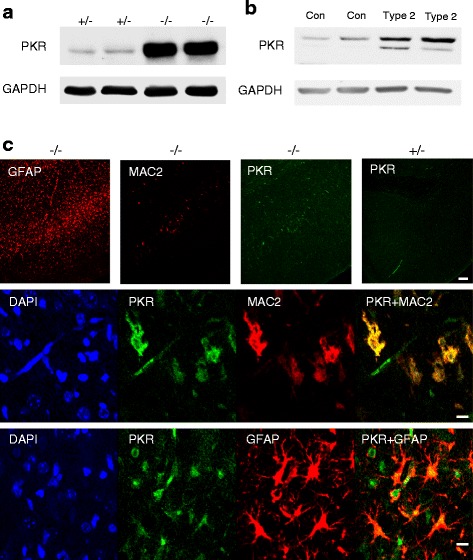
Elevation of PKR (*Eif2ak2*) in *Gba* ^−/−^ mice. **a** Western blot of homogenates (150 μg of protein) from the cortex of 21-day-old *Gba* ^−/−^ mice (*n* = 5). GAPDH was used as loading control. **b** Western blot of homogenates (50 μg of protein) from the cerebellum of two human patients who succumbed to type 2 GD compared to an age-matched control brain. **c** PKR levels in microglia/macrophages and in astrocytes in the *Gba* ^−/−^ brain. Immunofluorescence of cortical layer V in 16-day-old *Gba* ^−/−^ using anti-PKR (*green*), anti-MAC2 (*red*), or anti-GFAP (*red*) antibodies. *Upper panel* PKR staining is located in pathological areas as shown by staining of both MAC2 and GFAP. Scale bar, 100 μm. *Middle* and *lower panels* Double immunofluorescence of 16-day-old *Gba* ^−/−^ mice using either anti-PKR and anti-MAC2 (*middle panel*) or anti-PKR and anti-GFAP (*lower panel*) antibodies. PKR is in *green*, MAC2 and GFAP are in *red*, and areas of overlap are indicated on the *right*. Scale bar, 10 μm. Results are representative of three experiments

The double-stranded RNA-dependent protein kinase (PKR), encoding by the ISG [1] *Eif2ak2*, is a major antiviral effector due to its role in blocking protein synthesis by phosphorylating the α subunit of eukaryotic translation initiation factor 2α (eIF2α) [31]. PKR protein levels were highly elevated in the cerebral cortex of *Gba*^−/−^ mice (Fig. 3a), confirming the microarray results (Fig. 2b). PKR levels were also elevated in tissues from the two available cerebella obtained from human patients who succumbed to type 2 GD (Fig. 3b). Interestingly, PKR has recently been shown to play a role in programmed necrosis mediated by IFNs [32]. IFN induces association of PKR with receptor-interacting protein 1 (RIP1; RIPK1) to form assembly of the necrosome complex which contains RIP1, RIP3, and MLKL, and to initiate programmed necrosis and/or activation of the inflammasome; recently, we demonstrated that RIP3 is involved in nGD pathology [33]. PKR was selectively up-regulated in layer V of the cortex (Fig. 3c), another brain area which displays significant pathology in the *Gba*^−/−^ mouse, and MAC2-positive cells expressed high levels of PKR (Fig. 3c), similar to that observed for P-Stat2 (Fig. 2a) and for RIP3 [33]. PKR also co-localized with the astrocyte marker, GFAP (Fig. 3c).

### Ablation of the type I IFN receptor attenuates neuroinflammation but has no effect on the viability of nGD mice

**Fig. 4:**
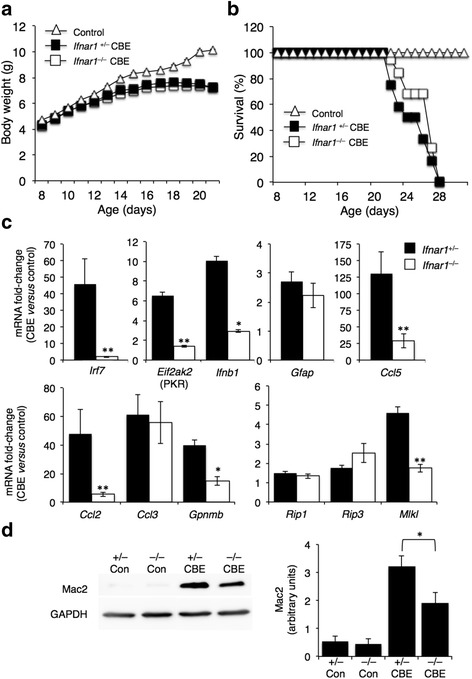
Effect of IFNAR on nGD progression and on down-stream signaling pathways. **a** Body weight of *Ifnar* ^+/−^ (*n* = 12) and *Ifnar* ^−/−^ (*n* = 19) mice treated with CBE (37.5 mg/kg per day) from 8 days of age or untreated mice (control, *n* = 9). Results are means ± s.e.m. **b** Kaplan-Meyer survival curves for *Ifnar* ^+/−^ (*n* = 12) and *Ifnar* ^−/−^ (*n* = 19) mice. **c** qPCR analysis of various genes in cortical homogenates from ~30-day-old *Ifnar* ^+/−^ (*n* = 2) and *Ifnar* ^−/−^ (*n* = 3) mice treated with CBE (37.5 mg/kg per day) from 8 days of age. Results are expressed as fold change of CBE-treated versus control and are means ± s.e.m. CT values were normalized to levels of HPRT. *p < 0.005, **p < 0.001. **d** *Left,* Western blots of homogenates (80 μg of protein) from cortex of ~30-day-old *Ifnar* ^+/−^ (+/−) and *Ifnar* ^−/−^ (−/−) (control) mice treated with or without CBE (+/− CBE, −/− CBE) from 8 days of age. Blots were probed with an anti-MAC2 antibody. Results are representative of three experiments for control mice and five for CBE treated mice. *GAPDH* was used as loading control. *Right* Densitometer analysis of the blots. *p < 0.05

To determine the role of the IFN-I receptor (IFNAR) in GD, IFN-I receptor-deficient (*Ifnar1*^−/−^) mice [34] were injected with conduritol B epoxide (CBE), an irreversible GCase inhibitor, which can be used to induce GD in mice with different severities depending on the dose used [35, 36]. Unlike *Rip3*^−/−^ mice, which develop GD upon CBE injection much more slowly than their littermate controls [33], no differences in the lifespan or in the weight of *Ifnar1*^−/−^ mice were observed upon CBE injection (Fig. 4a, b). However, the extent of elevation of genes down-stream to IFNAR, such as *Irf7*, *Ifnb1*, and *Eif2ak2*, was significantly lower in *Ifnar1*^−/−^ mice injected with CBE compared to *Ifnar1*^*+/−*^ mice. mRNA levels of other inflammatory markers that are elevated in nGD mice [12, 37] (i.e., *Ccl2* (MCP-1), *Ccl5* (RANTES), and glycoprotein non-metastatic B (GPNMB)) were significantly reduced in *Ifnar1*^−/−^ mice upon CBE injection (Fig. 4c), although no differences were observed in levels of *Ccl3* (MIP1α) and the astrocyte marker, *Gfap* (Fig. 4c). Levels of activated microglia/macrophages were higher upon CBE injection in both *Ifnar1*^−/−^ and *Ifnar1*^+/−^ mice (Fig. 4d), although blocking the type I IFN response caused a small but significant decrease in levels of activated microglia/macrophages (Fig. 4d), confirming a role for IFN in activation of the innate immune response in nGD.

mRNA levels of both *Rip1* and *Rip3* were up-regulated to a similar extent in both *Ifnar1*^−/−^ and *Ifnar1*^+/−^ mice upon CBE injection (Fig. 4c). Interestingly, mRNA levels of *Mlkl*, a crucial protein involved in necroptosis induction [38] and required for activity of the NLRP3 inflammasome, was significantly lower in *Ifnar1*^−/−^ mice injected with CBE (Fig. 4c), suggesting that *Mlkl* is an ISG.

### IFNβ production in neurons that accumulate GlcCer leads to ISG expression in neighboring microglia

**Fig. 5:**
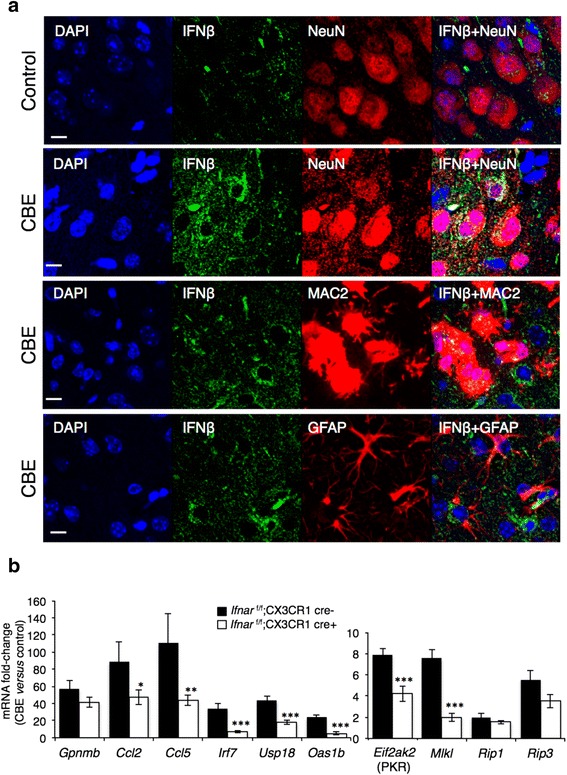
Induction of the IFN response in neurons and downstream signaling in microglia. **a** Elevation of IFNβ in neurons in the cerebral cortex of 18-day-old CBE-treated mice (100 mg/kg, from 8 days of age) compared to untreated mice (control). Double immunofluorescence using either anti-GFP (IFNβ, *green*) and anti-NeuN (*red*), anti-GFP and anti-MAC2 (*red*), or anti-GFP and anti-GFAP (*red*) antibodies and the nuclear marker, DAPI (*blue*). Scale bar, 10 μm. Results are representative of two experiments. **b** qPCR analysis of various genes in cortical homogenates from 18-day-old *Ifnar* ^flox/flox^; CX3CR1-Cre + (*n* = 4) and *Ifnar* ^flox/flox^; CX3CR1-Cre- (*n* = 4) mice treated with CBE (100 mg/kg per day) from 8 days of age for 10 days. Results are expressed as fold change of CBE-treated versus control (*n* = 3–4) mice and are means ± s.e.m. CT values were normalized to levels of HPRT. *p < 0.05, **p < 0.01, ***p < 0.001

To determine in which cell types IFNβ was elevated in nGD mice, *Ifnb1*^*tm1Lky*^/J mice, which co-express *Ifnb1* and EYFP from the *Ifnb1* locus, were injected with CBE. IFNβ immunoreactivity was significantly elevated in layer V of the cerebral cortex of CBE-treated mice (Fig. 5a), mainly in neurons and occasionally in microglia, but no elevation was detected in astrocytes. To determine the contribution of microglia to ISG expression down-stream to IFNAR, nGD was induced by CBE injection in *Ifnar*^flox/flox^; CX3CR1-Cre mice [39, 40], which lack the IFN receptor in mononuclear phagocytic cells (i.e. microglia in the brain). While mRNA levels of *Gpnmb*, *Rip1*, and *Rip3* were up-regulated to a similar extent as in control (*Ifnar*^flox/flox^) mice upon CBE treatment, levels of *Ccl2*, *Ccl5*, *Irf7*, *Eif2ak2* (PKR), *Usp18*, *Oas1b*, and *Mlkl* were significantly lower in the *Ifnar*^flox/flox^; CX3CR1-Cre mice (Fig. 5b), indicating that their transcription is down-stream to IFNAR in microglia in nGD brain.

### Distinct inflammatory profiles in brains from different LSD mouse models

Brain inflammation and microglial activation are shared features of many LSDs [13, 14, 41–43]. Thus, we examined whether IFNs and antiviral ISGs are up-regulated in other LSDs which present significant brain inflammation. Expression levels of the astrocyte marker, *Gfap*, and the myeloid-lineage cell marker, *Adgre1* (F4/80), as well as the inflammatory chemokines *Ccl3*, *Ccl5, Gpnmb, Il-1β*, and *Tnfα* were up-regulated in cerebral hemispheres obtained from the most severe stage of mouse models of nGD [21], Krabbe’s disease [44], Niemann-Pick type C1 disease (NPC1) [5] and Sandhoff disease [45], indicative of neuroinflammation (Table 2). However, levels of *Ifnb1* were only significantly up-regulated in cerebral hemispheres from nGD mice, and to a small extent in Krabbe mice; no significant elevation was detected in either NPC1 or in Sandhoff disease (Table 2), suggesting that the antiviral response is elevated specifically in diseases in which simple glycosphingolipids are elevated. Genes related to the RIPK pathway were likewise only elevated in GD and Krabbe’s disease (Table 2).

**Table 2:** qPCR analysis of genes in cerebral hemispheres obtained from the end-stage of various LSDs

Gene	nGD		Krabbe		NPC1		Sandhoff	
	Fold change (−/− vs*.* +/−)	*p* value	Fold change (−/− vs. +/−)	*p* value	Fold change (−/− vs. +/−)	*p* value	Fold change (−/− vs. +/−)	*p* value
Inflammation								
*Gfap*	**6.8 ± 1.6**	<0.005	**2.6 ± 0.8**	<0.05	**2.1 ± 0.6**	0.059	**2 ± 0.5**	<0.05
*Adgre1* (F4/80)	**6.6 ± 0.4**	<0.001	**3.9 ± 0.7**	<0.005	**3.6 ± 1.1**	<0.05	**2.7 ± 0.4**	<0.05
*Gpnmb*	**78.4 ± 15.6**	<0.001	**77.2 ± 45.4**	<0.05	**12.6 ± 4.9**	<0.05	**2.6 ± 0.4**	<0.005
*Tnf a*	**11.2 ± 2.5**	<0.005	**9.3 ± 5.12**	<0.05	1.7 ± 0.3	<0.05	1.6 ± 0.2	<0.05
*Il-1β*	**7.5 ± 4.6**	<0.005	**4.4 ± 1.0**	<0.005	**5.8 ± 1.4**	<0.05	**2.8 ± 0.1**	<0.005
*Ccl2*	**129.2 ± 46.3**	<0.005	**25.4 ± 16.1**	<0.05	**3.9 ± 1.2**	<0.05	**3.5 ± 1.5**	<0.05
*Ccl3*	**371.3 ± 131.7**	<0.05	**18.0 ± 9.3**	<0.001	**11.8 ± 3.5**	<0.05	**6.7 ± 1.3**	<0.001
*Ccl5*	**240 ± 19.3**	<0.001	**11.3 ± 2.2**	<0.001	**4.7 ± 1.2**	<0.05	**2 ± 0.3**	<0.001
*Il-10*	1.8 ± 0.4	<0.05	2.0 ± 0.6	ns	0.9 ± 0.2	ns	1.1 ± 0.2	ns
*Il-6*	**22.1 ± 7.0**	<0.005	**7.4 ± 3.1**	<0.05	1.7 ± 0.8	ns	1.1 ± 0.3	ns
Anti-viral response								
*Ifnα*	1.7 ± 0.8^a^	ns	2.0 ± 1.3	ns	0.9 ± 0.5	ns	1.5 ± 0.7	ns
*Ifnβ*	**3.9 ± 1.7**	<0.05	2.1 ± 1.7	ns	1 ± 0.5	ns	1 ± 0.4	ns
*Irf7*	**130.9 ± 18.4**	<0.001	**7.3 ± 2.9**	<0.05	**4.6 ± 0.8**	<0.001	1.6 ± 0.2	<0.05
*Usp18*	**104.4 ± 13.3**	<0.001	**11.2 ± 2.4**	<0.001	**7.1 ± 0.5**	<0.001	1.6 ± 0.2	<0.05
*Oas1b*	**22.6 ± 5.6**	<0.001	**5. 7 ± 2.1**	<0.05	2.6 ± 1.2	ns	1.1 ± 0.4	ns
*Eif2ak2*	**11.7 ± 1.1**	<0.001	**3.5 ± 1**	<0.05	1.9 ± 0.3	<0.05	1.4 ± 0.1	<0.05
RIPK pathway								
*Rip1*	**2.3 ± 0.4**	<0.005	**2 ± 0.36**	<0.05	1.4 ± 0.2	<0.05	0.9 ± 0.04	ns
*Rip3*	**9.1 ± 0.9**	<0.001	**2.3 ± 0.27**	<0.05	1.5 ± 0.2	<0.05	0.7 ± 0.1	ns
*Mlkl*	**9.4 ± 1.8**	<0.001	**4.74 ± 1.46**	<0.05	1.8 ± 0.2	0.00898	1.2 ± 0.3	ns

Results are expressed as fold change of −/− versus control mice and are means ± s.e.m. CT values were normalized to levels of HPRT. Genes that were elevated >2-fold with a *p* value of <0.05 are in bold. nGD mice (*Gba* ^flox/flox^; nestin-Cre mice, 3 weeks of age, *n* = 4); Krabbe’s disease (*Galc* ^−/−^ mice, 5 weeks of age, *n* = 3); Niemann-Pick type C1 disease mice (NPC1^−/−^), 10 weeks of age, *n* = 3–4; Sandhoff disease (*HexB* ^−/−^ mice) 17 weeks of age, *n* = 2–3

^a^It should be noted that there is a significant difference in the extent of elevation of mRNA levels depending on whether a pathological area of the brain was used compared to half cerebral hemispheres. For instance, *Ifnα* was elevated 2.1-fold in layer V of the cortex, but 1.7-fold in a cerebral hemisphere. However, even when using whole cerebral hemispheres, it is apparent that the extent of the inflammatory response is much higher in nGD compared to the other LSDs

## Discussion

Type I IFNs are mainly recognized as part of the host response to viral and bacterial infection. Much less is known about the role of the type I IFN response under conditions of sterile inflammation, particularly in the CNS [3–6]. Activation of this response in the absence of infection can either be detrimental, as in AD, ALS, and AGS, or beneficial, as in multiple sclerosis. Interestingly, depletion of IFNβ causes spontaneous neurodegeneration resembling sporadic Lewy body and PD [46]. In the current study, we demonstrate massive induction of the type I IFN response in nGD brain at a pre-symptomatic stage, suggesting a key role for the antiviral response in the initial stages of brain pathology.

**Fig. 6:**
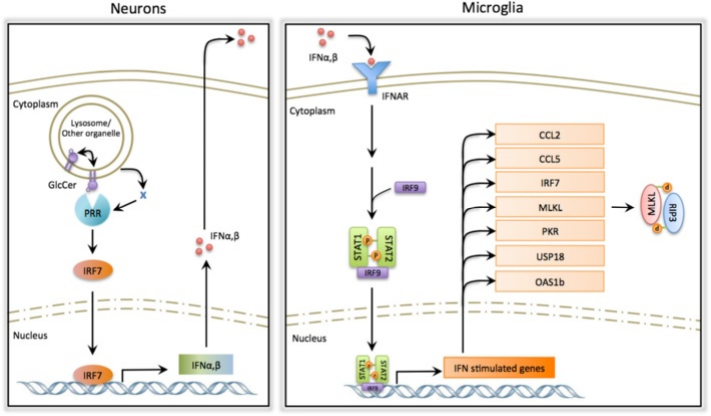
Proposed mechanism by which type I IFN is activated in nGD. *Left* Induction of the IFN response in neurons upon GlcCer accumulation. Elevated levels of GlcCer are sensed directly or indirectly by a PRR resulting in activation of the antiviral response and production of IFNα and IFNβ. IFN is secreted from neurons and engages IFNAR in neighboring cells, i.e., microglia. *Right* Upon engagement, IFNAR activates phosphorylation, dimerization, and nuclear translocation of STAT proteins, which subsequently induce the expression of ISGs, among them MLKL, which forms the necrosome complex together with phosphorylated RIP3

The mechanism by which the immune response is triggered in nGD is not known. Importantly, in the *Gba*^flox/flox^; nestin-Cre mouse, microglia do not accumulate GlcCer [21] and their activation cannot therefore be due to intrinsic GlcCer accumulation, but must rather be a response to extrinsic signals from other cells that do accumulate GlcCer, such as neurons and/or astrocytes. PRRs, ISGs, and other components of innate immunity are expressed in astrocytes, microglia, and neurons [47, 48], and our data is consistent with the idea that the initial activation of the immune response is up-stream to IFNAR in microglia. A remaining challenge is to determine if the immune response is triggered by PRRs which directly sense increased GlcCer levels or by PRRs that sense known PAMPs/DAMPs such as dsRNA, cytosolic DNA, glycoproteins, and nucleic acids, which for unknown reasons may be altered in nGD (Fig. 6). Constitutive up-regulation of type I IFN production was found in a group of monogenic diseases characterized by neurological and dermatological features, known as type I interferonopathies, which result from accumulation of endogenous nucleic acid products, which are subsequently sensed as non-self by the innate immune machinery [8]. Cross-talk between changes in lipid composition and TLR signaling was recently demonstrated, with GD fibroblasts displaying a hyper-inflammatory response to TLR-induced IL-6 release [49]. This, together with our data, suggests a scenario by which GlcCer is recognized as a DAMP upon its accumulation in nGD leading to IFN induction and secretion. Subsequently, IFN activates surrounding microglia via binding to IFNAR, resulting in the phosphorylation of STAT and induction of ISGs [27]. Neurons from distinct brain regions have unique innate immune signatures which determine their susceptibility to viral infection [50], and we speculate that different neuronal populations have different immunological properties which might explain their selective vulnerability to GlcCer accumulation in nGD [16] and to other lipids and/or substrates in other LSDs.

**Fig. 7:**
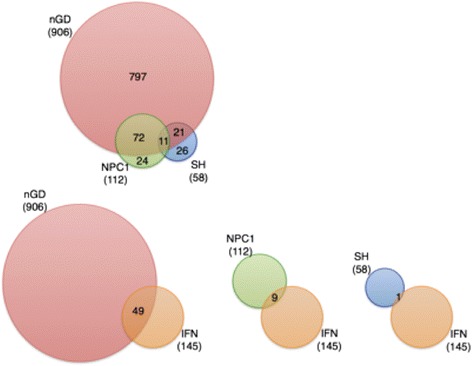
Gene changes in LSDs. *Upper panel* Venn diagram of differentially-expressed genes in spinal cord of Sandhoff disease, brain of NPC1 disease, and the VPM/VPL of nGD. *Lower panel* Venn diagram of differentially expressed genes related to the type I IFN pathway to up-regulated genes in other diseases. The enrichment IFN signaling lists were obtained from GSEA (REACTOME_INTERFERON_SIGNALING). Overlapping regions were drawn to scale and the number of shared and unique genes listed. Overlap was visualized using BioVenn

Brain inflammation is a universal feature of many neurodegenerative diseases, including LSDs with brain involvement. The inflammatory response can take many guises, being initiated in response to different stimuli, can be chronic or acute, beneficial or detrimental, all of which can be mediated by different inflammatory factors. Each LSD has its own pattern of accumulation of storage material and its own pattern of neuroinflammation [42]. It is notable that of the four LSDs that we studied, nGD gave the most severe inflammatory response, although this might, at least in part, be due to the rapid onset of disease symptoms in the *Gba*^flox/flox^; nestin-Cre mouse compared to the other mouse models examined. The *Gba*^flox/flox^; nestin-Cre mouse displayed by far the most robust type 1 IFN response, as exemplified by highly elevated levels of *Irf7* and *Usp18*. Ours is of course not the first study to examine patterns of gene activation in the brains of LSD mice or patients, but lack of consistency in the age of the brain material used, and in the age of animals, renders quantitative comparison difficult. Nevertheless, some information can be gleaned from such a comparison. For instance, in a gene microarray analysis using spinal cord tissue from 4-month-old Sandhoff disease mice [51], 58 genes were up-regulated, of which 32 are common to nGD, although only one of these is involved in IFN signaling (Fig. 7 and Table 3). A microarray analysis on *Npc1*^−/−^ mouse brain at different stages of disease severity [52] revealed up-regulation of 112 genes, of which 88 were also up-regulated in nGD, and 9 of these are in the IFN signaling pathway. IFNβ secretion and increased STAT levels were demonstrated in cultured human NPC fibroblasts [53]. The similarity of the lipids that accumulate in nGD (GlcCer/GlcSph), Krabbe (galactosylsphingosine), and NPC (in which small but significant amounts of GlcCer accumulate in addition to the primary storage material, cholesterol) suggest that more complex GSLs such as ganglioside GM2, which accumulate in Sandhoff disease, cannot trigger the IFN response, whereas the more simple GSLs do trigger this response, although to a different extent.

**Table 3:** Comparison of gene arrays for NPC1, Sandhoff, and nGD

	Sandhoff [51]	NPC [52]	nGD
Tissue	Spinal cord	Brain	VPM/VPL
Age/disease severity	4 months old (late-symptomatic stage)	20 to 80 days old (asymptomatic-, early-, and late-symptomatic stages)	14 days old (asymptomatic stage)
Analysis cut-off	2-fold	1.5-fold	1.5-fold
Number of up-regulated genes	58	112	907
Number (and percent) of up-regulated genes common to nGD	32 (55.2 %)	88 (78.5 %)	
Number (and percent) of IFN-related genes among the up-regulated genes	1 (1.7 %)	9 (8 %)	49 (5.4 %)
Percentage of IFN-related gene out of total of 145 IFN-related genes	0.70 %	6.20 %	33.80 %

We recently demonstrated a critical role for RIP3 in nGD pathology [33], although we have so far not been able to determine the precise mechanistic role of RIP3. Of some interest is the observation that viral and bacterial pathogens induce programmed necrosis via a RIP3-dependent cell death mechanism [54–57], which can involve type I IFN [58]. Unlike RIP3, ablating the type 1 IFN response, using the *Ifnar1*-deficient mouse, did not alleviate disease symptoms in nGD mice, and moreover, mRNA levels of *Rip1* and *Rip3* were essentially unaltered. In contrast, expression of *Mlkl*, the key down-stream player to RIP1-RIP3 in programmed necrosis, was significantly lower in *Ifnar1*^−/−^ mice, indicating that *Mlkl* is a novel ISG. We would like to verify this by examining the phosphorylation state of RIP3 and MLKL, but unfortunately, no commercial antibodies against the phosphorylated forms of mouse RIP3 and mouse MLKL are currently available [59].

At this stage, we do not know if activation of type I IFN signaling in nGD brain is beneficial or detrimental. The lack of benefit upon induction of nGD in *Ifnar1*^−/−^ mice is somewhat surprising and might be due to activation of a compensatory inflammatory mechanism upon interfering with IFN signaling pathways; alternatively, endogenous activation of proteins that block interferon signaling, such as *Usp18* (Fig. 4), might alleviate any beneficial effect of IFN. *Usp18* is arguably the ISG with the most important role in establishing and maintaining long-term desensitization to type I IFN signaling [30]. This desensitized state allows cells to recover from IFN signaling, whereas dysregulation of IFN production and signaling manifests in autoimmune disorders such as systemic lupus erythematosus and Sjögren’s syndrome [60]. Finally, we cannot exclude the possibility that mice treated with CBE succumb to systemic disease, rather than a disease of the CNS, and therefore, amelioration of neurological disease might not be sufficient to alleviate disease symptoms in the case of the *Ifnar1*^−/−^ mouse.

## Methods

### Mice

*Gba*^flox/flox^; nestin-Cre mice were used as a genetic model of nGD [21]. *Gba*^flox/flox^ mice were crossed with *Gba*^flox/wt^; nestin-Cre mice to generate *Gba*^flox/flox^; nestin-Cre mice (referred to as *Gba*^−/−^ mice) and *Gba*^flox/wt^; nestin-Cre mice (referred to as *Gba*^+/−^ mice), which served as healthy controls since they do not show any overt pathology [22]. IFN-I receptor-deficient (*Ifnar1*^−/−^) mice [34] were backcrossed with C57BL/6 mice to generate *Ifnar1*^*+/−*^ mice. *Ifnar1*^*+/−*^ mice were crossed with *Ifnar1*^−/−^ mice to generate *Ifnar1*^*+/−*^ and *Ifnar1*^−/−^ littermates. *Ifnar1*^*+/−*^ and *Ifnar1*^−/−^ mice were injected daily i.p. with 37.5 mg/kg body weight CBE, an irreversible GCase inhibitor [36], from 8 days of age. *Ifnb1*^*tm1Lky*^/J mice were obtained from Jackson and were injected daily i.p. with 100 mg/kg body weight CBE from 8 days of age. *Ifnar*^flox/flox^ mice [61] were crossed with CX3CR1-Cre mice [40] to generate *Ifnar*^flox/wt^; CX3CR1-Cre mice. *Ifnar*^flox/flox^; CX3CR1-Cre mice were crossed with *Ifnar*^flox/flox^ mice to generate *Ifnar*^flox/flox^; CX3CR1-Cre and *Ifnar*^flox/flox^ littermates. *Ifnar*^flox/flox^; CX3CR1-Cre and *Ifnar*^flox/flox^ mice were injected daily i.p. with 100 mg/kg body weight CBE from 8 days of age. Mice deficient in the β subunit of β-hexosaminidase A and B were used as mouse model of Sandhoff disease [45]. Brains from a mouse model of Krabbe’s disease [44], defective in β-galactocerebrosidase (the Twitcher mouse), were provided by Dr. Timothy M. Cox (University of Cambridge). Brains from Niemann-Pick disease type C1 mice, defective in the *Npc1* gene [62], were provided by Dr. Nick Platt (Oxford University). 

### Human brain tissue

A control human brain from an infant who died at birth was provided by the University of Miami Brain and Tissue Bank for Developmental Disorders. The control brain was frozen within 6 and 26 h of death. Brains from Gaucher patients were obtained postmortem with informed consent between 7 and 22 h after death [63]. After removal, brains were frozen on dry ice.

### Gene expression profiling microarray analysis

Mice were sacrificed and their brains removed and placed on a Young Mouse Brain Slicer Matrix (BSMYS001-1, Zivic instruments, Pittsburgh, PA, USA). Single-edge razor blades were inserted into the matrix to generate 1-mm coronal sections. Sections containing the VPM/VPL were snap-frozen on dry ice and a 17-g (1 mm diameter) blunt needle attached to a syringe was used to remove the VPM/VPL; the cerebral cortex was separated using a spatula. Each sample was placed in a microcentrifuge tube, snap-frozen in liquid N_2_, and stored at −80 °C.

Total RNA was isolated using the RNeasy Mini Kit (QIAGEN GmbH, Hilden, Germany) according to manufacturer’s instructions, which included DNase treatment and addition of β-mercaptoethanol. A microarray experiment was performed on 100-ng RNA per sample purified from the VPM/VPL region of *Gba*^+/–^ and *Gba*^−/−^ mice at 14 days (*n* = 3) of age. Purified RNA was reverse-transcribed and amplified using an Ambion WT expression kit and labeled with an Affymetrix GeneChip® WT Terminal Labeling kit. Labeled cDNA was hybridized to Affymetrix Mouse Gene 1.0 microarrays according to manufacturer’s instructions. Microarrays were scanned using a GeneChip® scanner 30007G and statistical analysis performed using Partek® Genomics Suite software (Partek Inc., St. Louis, MI, USA). For each age group, CEL files (containing raw expression measurements) were imported to Partek GS. The data was processed and normalized using the RMA (Robust Multichip Average) algorithm [64] with GC correction, and a three-way ANOVA model was used to identify differentially expressed genes; fold-changes were calculated. Analysis of *Gba* gene probe sets verified that the mRNA expression of *Gba* flanked exons [21] was reduced in *Gba*^−/−^ samples. Gene lists were created by filtering the genes based on an absolute linear fold change ≥1.5, *p* ≤ 0.05, and signal above background in at least one microarray (log2 intensity ≥6) (Additional file 1: Table S1). The gene lists were analyzed for enriched pathways using GSEA (www.broadinstitute.org/gsea), Ontologizer (Elim algorithm) [23], DAVID [65] against the KEGG pathways database, and IPA (Ingenuity® Systems, www.ingenuity.com). The enrichment of the lists was tested against the expressed genes (above background signal) when implementing the Ontologizer tool. Microarray data were deposited in the Gene Expression Omnibus (GEO) database, www.ncbi.nlm.nih.gov/geo (accession no. GSE46866). The BioVenn tool (http://www.cmbi.ru.nl/cdd/biovenn/) [66] was used to identify common and exclusively expressed genes between groups.

### qPCR

cDNA synthesis was performed using the Reverse-iT First-Strand Synthesis Kit (Thermo Fisher Scientific, Surrey, UK) using random decamers. cDNA products were stored at −20 °C. qPCR was performed using PerfeCTa SYBR Green FastMix (Quanta BioSciences, Gaithersburg, MD, USA) and an ABI Prism 7300 Sequence Detection System (Applied Biosystems, Foster City, CA, USA). The primer concentration was 13 nM in a reaction volume of 20 μl and cDNA equivalent to 2–20 ng of total RNA, or 300 ng for *Ifnb1* and *Ifna2*. Each reaction was performed in triplicate. The thermal cycling parameters were as follows: step 1, 95 °C for 10 min; step 2, 95 °C for 15 s, 60 °C for 30 s, and 68 °C for 30 s. Step 2 was repeated for 40 cycles and was followed by a dissociation step. Fold change in mRNA levels was calculated using the comparative cycle threshold method using TATA box binding protein (TBP) or hypoxanthine phosphoribosyltransferase 1 (HPRT) for normalization. *p* values were calculated using a two-tailed, two-independent sample Student’s *t* test. Primers for *Ifnb1*, *Ifna2*, *Il-1β*, and *Il-10* were from QIAGEN. Primers sequences are in Table 4.

**Table 4:** Primers used for qPCR

Gene name	Primer sequence
Tbp	F: 5′-TGCTGTTGGTGATTGTTGGT-3′
	R: 5′-CTGGCTTGTGTGGGAAAGAT-3′
Hprt	F: 5′-TGCTCGAGATGTCATGAAGG-3′
	R: 5′-AATCCAGCAGGTCAGCAAAG-3′
Lgals3 (Mac2)	F: 5′-CACTGACGGTGCCCTATGAC-3′
	R: 5′-AACAATCCTGTTTGCGTTGGG-3′
Kcnk4	F: 5′-GCAGGCTCAGAAGAAAATGG-3′
	R: 5′-TGGTCCCTCAGAAACTGGTC-3′
Sptssb	F: 5′-TCATTCTAAGGCAGGAGACGA-3′
	R: 5′-AAGCTGGGAAAAGTCTGCCT-3′
Ryr3	F: 5'-GGTGGGCTATTACTGCCTGA-3'
	R: 5'-CATCCTCAGATGGCTGTTCA -3'
Gfap	F: 5′-TAGTCCAACCCGTTCCTCCA-3′
	R: 5′-CCAGTTGTCGACTAGGACCG-3′
Irf7	F: 5′-CAATGGCTGAAGTGAGGGGG-3′
	R: 5′-GACCGAAATGCTTCCAGGGT-3′
Eif2ak2 (PKR)	F: 5′-GATGGAAAATCCCGAACAAGGAG-3′
	R: 5′-AGGCCCAAAGCAAAGATGTCCAC-3′
Ccl5	F: 5′-TGCCTACCTCTCCCTCGCGC-3′
	R: 5′-GGCACACACTTGGCGGTTCCT-3′
Ccl2	F: 5′-TCACCTGCTGCTACTCATTCACCA-3′
	R: 5′-AGCACAGACCTCTCTCTTGAGCTT-3′
Ccl3	F: 5′-TTTTGAAACCAGCAGCCTTT-3′
	R: 5′-CTCAAGCCCCTGCTCTACAC-3′
Gpnmb	F: 5′-AGCACAACCAATTACGTGGC-3′
	R: 5′-CTTCCCAGGAGTCCTTCCA-3′
Rip1	F: 5′-AGTCGAGACTGAAGGACACAGCACT-3′
	R: 5′-TCCAGCAGGTCACTGGATGCCAT-3′
Rip3	F: 5′-CTTGAACCCTCCGCTCCTGC-3′
	R: 5′-CGGCTCACCAGAGGAACCGCAT-3′
Mlkl	F: 5′-TCACAGATCTCCAGTTACCATC-3′
	R: 5′-ACGCAAGATGTTGGGAGAATCG-3′
Tnfα	F: 5′-CTTGTGGCAGGGGCCACCAC-3′
	R: 5′-CCATGCCGTTGGCCAGGAGG-3′
Usp18	F: 5′-CAGGAGTCCCTGATTTGCGT-3′
	R: 5′-GGGCTGGACGAAACATCTCA-3′
Oas1b	F: 5′-GCGTAGGCCTGTTATGCTCT-3′
	R: 5′-CCACTGGGCTCACAGGAAAA-3′

### ELISA

Frozen cerebral brain tissues were homogenized in 0.1 M phosphate buffer (PBS) supplemented with a protease inhibitor cocktail (1:200, Sigma). Samples were sonicated and centrifuged at 14,000*g*_av_ for 10 min at 4 °C, and the supernatant was collected. Protein was quantified using the BCA protein assay reagent (Pierce Chemical Co., Rockford, Illinois, USA). Homogenates containing ~3–4 mg of protein were applied to a VeriKine™ mouse Interferon Alpha ELISA kit (PBL Interferon Source, Piscataway, NJ, USA), ~2 mg of protein were applied to Verikine-HS™ mouse Interferon Beta Elisa kit (PBL Interferon Source), and 1 mg was applied to a VeriKine™ Human Interferon Beta ELISA kit (PBL Interferon Source, Piscataway, NJ, USA) according to manufacturer’s guidelines.

### Immunohistochemistry

Brains were removed and immersion fixed in 2.5 % paraformaldehyde in 0.1 M PBS, pH 7.4 for 2 days, embedded in paraffin blocks, and 4-μm coronal sections prepared on Superfrost + slides. Sections were deparaffinized and rehydrated prior to further use. Antigen retrieval was performed using 10 mM citric acid (pH 6.0) for 10 min at ~100 °C. Sections were blocked using 20 % normal horse serum (NHS) (Vector, Burlingame, CA, USA) and 0.3 % Triton X-100. Sections were incubated with either rabbit antiphospho-Y690-Stat2 (1:50, Abcam, Cambridge, MA), mouse anti-NeuN (1:50, Chemicon, Temecula, CA, USA), rat anti-MAC2 (1:250, Cedarlane, Ontario, Canada), mouse anti-PKR (1:100, Santa Cruz), rabbit anti-GFAP (1:100, Dako), or with a goat anti-GFP antibody (Biotin, 1:100, Abcam, Cambridge, MA). Antibody mixtures were diluted in 2 % normal horse serum (NHS) containing 0.3 % Triton X-100. Sections were incubated with the first antibodies overnight at room temperature. For P-Stat2 detection, sections were incubated with a biotinylated donkey antirabbit secondary antibody (1:200, Jackson, West Grove, PA, USA) and for PKR with a biotinylated donkey antimouse secondary antibody (1:200, Jackson, West Grove, PA, USA) for 2 h followed by Cy3-conjugated streptavidin (1:200, Jackson) for 1 h. NeuN-stained samples were incubated with a Cy2-conjugated donkey antimouse antibody (1:200, Jackson ImmunoResearch), MAC2 with a Cy2-conjugated donkey antirat antibody (1:200, Jackson ImmunoResearch), and GFAP with a Cy2-conjugated donkey antirabbit antibody (1:200, Jackson ImmunoResearch) for 1 h. Counterstaining was performed with DAPI (Molecular Probes, Eugene, OR, USA) (1:1000, 4 min), sections were rinsed with PBS, mounted with Flouromount-G (SouthernBiotech), and coverslipped.

### Protein extraction and Western blotting

Homogenates were prepared as described [67]. Blots were incubated with the following antibodies: mouse anti-PKR (1:1,000, Santa Cruz), rat anti-MAC2 (1:1,000, Cell Signaling), and a mouse anti-GAPDH (1:10,000, Chemicon), followed by incubation with a horseradish peroxidase-conjugated secondary antibody (antimouse, Jackson ImmunoResearch). Bound antibodies were detected using the SuperSignal West Pico chemiluminescent substrate (Thermo Scientific).

## Conclusions

The type I IFN response appears to be a significant player in the development of nGD pathology and possibly in other LSDs in which simple GSLs accumulate. Our data support the developing notion that the IFN signaling pathway (Fig. 6) plays a vital role during the sterile inflammation that often occurs during chronic neurodegenerative diseases in which neuroinflammation is present.

## Ethics approval

Mice were maintained under specific pathogen-free conditions and handled according to protocols approved by the Weizmann Institute Animal Care Committee according to international guidelines.

## Consent for publication

Not applicable.

## Availability of data and materials

All material used in this manuscript will be made available to researchers subject to confidentiality.

## Additional file

Additional file 1: List of the differentially-expressed transcripts. (XLSX 330 kb)

## Abbreviations

GD: Gaucher disease
GCase: acid-β-glucosidase
GlcCer: glucosylceramide
Ifn: interferon
Irf: interferon regulatory factor
ISG: interferon-stimulated gene
LSD: lysosomal storage disease
nGD: neuronopathic Gaucher disease
qPCR: quantitative polymerase chain reaction
STAT: signal transducers and activators of transcription
VPM/VPL: ventral posteromedial/posterolateral
